# Exploring the efficacy and safety of a novel standardized ashwagandha (*Withania somnifera*) root extract (Witholytin®) in adults experiencing high stress and fatigue in a randomized, double-blind, placebo-controlled trial

**DOI:** 10.1177/02698811231200023

**Published:** 2023-09-23

**Authors:** Stephen J Smith, Adrian L Lopresti, Timothy J Fairchild

**Affiliations:** 1 Clinical Research Australia, Perth, WA, Australia; 2 College of Science, Health, Engineering and Education, Murdoch University, Perth, WA, Australia

**Keywords:** Ashwagandha, *Withania somnifera*, stress, fatigue, hormones, testosterone

## Abstract

**Background:**

Stress is a state of homeostasis in the body being challenged, resulting in a systemic response. It has become more prevalent in recent years and affects mental and physical health.

**Aims:**

Evaluate the effects of ashwagandha on stress, fatigue, and sex hormones in overweight or mildly obese men and women with self-reported stress and fatigue.

**Methods:**

Two-arm, parallel-group, 12-week, randomized, double-blind, placebo-controlled trial on overweight or mildly obese men and women aged 40–75 years, supplementing with 200 mg of an ashwagandha root extract (Witholytin®) twice daily.

**Results/Outcomes:**

Supplementation with ashwagandha was associated with a significant reduction in stress levels based on the Perceived Stress Scale (primary outcome); however, the improvements were not significantly different to the placebo group (*p* = 0.867). Based on the Chalder Fatigue Scale, there was a statistically significant reduction in fatigue symptoms in the ashwagandha group compared to the placebo group (*p* = 0.016), and participants taking ashwagandha also experienced a significant increase in heart rate variability (*p* = 0.003). However, there were no significant between-group differences in other self-report outcome measures. In the men taking ashwagandha, there was a significant increase in the blood concentrations of free testosterone (*p* = 0.048) and luteinizing hormone (*p* = 0.002) compared to the placebo group.

**Conclusions/Interpretation:**

The results of this study suggest that in overweight middle-to-older age adults experiencing high stress and fatigue, compared to the placebo, ashwagandha did not have a significantly greater impact on perceived stress levels. However, based on secondary outcome measures, it may have anti-fatigue effects. This may be via its impact on the autonomic nervous system. However, further research is required to expand on these current findings.

## Introduction

Stress is defined as the state of homeostasis in the body being challenged, resulting in a systemic response (Lu et al., 2021). It has become more prevalent in recent years, with almost one in three people experiencing some degree of stress or anxiety (Salari et al., 2020). Stress can significantly affect mental and physical health (Cohen et al., 2007) and increases the risk of future morbidity and mortality (Pedersen et al., 2011). Moreover, a systematic review investigated the evidence from longitudinal and cross-sectional studies and concluded that stress is inversely associated with quality of life (Hohls et al., 2021). In another review, the consequences of psychological stress on aging were examined, and it was concluded that it may compromise healthy aging (Moreno-Villanueva and Burkle, 2015). Given the prevalence of stress, its association with mental and physical health, and its effect on quality of life, identifying strategies to reduce its impact is prudent.

Fatigue, or lack of energy/vitality, is another common problem, with approximately 20% to 40% of adults presenting with symptoms associated with fatigue (Lerdal et al., 2005). Similar to stress, fatigue is associated with future morbidity and mortality (Appels and Mulder, 1988); functional limitations, disability, and risk of hospitalization (Avlund, 2010); and a reduced quality of life (Yoo et al., 2018). Several studies indicate that fatigue increases with advancing age (Malak et al., 2021; Zengarini et al., 2015). Moreover, stress, fatigue, and advancing age are all associated with changes in sex hormone concentrations in both men and women (Pletzer et al., 2021), with estrogen and testosterone generally decreasing in women and men, respectively; dehydroepiandrosterone sulphate (DHEA-S) decreasing in both sexes; and luteinizing hormone (LH) increasing in both sexes (Chahal and Drake, 2007). However, the data regarding the effects of stress on sex hormones are inconsistent, with researchers concluding that the stress response is not restricted to the hypothalamus-pituitary-adrenal (HPA) axis and cortisol concentrations (Pletzer et al., 2021). Sex hormones have many important roles in the body, including the production of red blood cells (Beggs et al., 2014), promotion of skeletal integrity (Oury, 2012), modification of body composition (Finkelstein et al., 2013), and enhanced sexual responsiveness (Rastrelli et al., 2018). Therefore, identifying novel ways to improve endogenous sex hormone production in both men and women as they age is worthwhile.

Herbal or plant preparations that assist the body in tolerating, resisting, and adapting to stressors are collectively known as adaptogens. They have demonstrated anti-fatigue, antidepressant, anxiolytic, stress-reduction, and healthy-aging effects (Panossian, 2017; Panossian and Wikman, 2009, 2010). There are numerous examples of adaptogens, including *Withania somnifera* (ashwagandha), *Rhodiola rosea*, *Tribulus terrestris*, *Panax ginseng* (Korean ginseng), *Eleutherococcus senticosus* (Siberian ginseng), and *Bacopa monnieri*, with ashwagandha being one of the most commonly used adaptogens in Ayurvedic medicine (Panossian et al., 2021), as well as one of the most extensively researched herbs (Provino, 2010). Several clinical trials have demonstrated ashwagandha’s efficacy in reducing stress levels (Chandrasekhar et al., 2012; Choudhary et al., 2017; Gopukumar et al., 2021; Salve et al., 2019). A meta-analysis examining the effects of ashwagandha on physical performance, including fatigue, concluded that ashwagandha may also reduce fatigue symptoms (Bonilla et al., 2021). Therefore, this study aimed to evaluate the impact of supplementation with ashwagandha for 12 weeks on stress (primary outcome), fatigue, and general well-being in adults. Moreover, blood markers were measured over time to help understand the mechanisms of action associated with ashwagandha supplementation. A systematic review identified ashwagandha as one of the most effective herbal extracts for increasing testosterone concentrations in men (Smith et al., 2021), and in a study on women experiencing perimenopausal symptoms, ashwagandha increased estradiol concentrations and decreased LH concentrations (Gopal et al., 2021). In previous trials, ashwagandha has also altered thyroid hormone concentrations (Sharma et al., 2018), stabilized blood glucose concentrations (Durg et al., 2020), and reduced oxidative stress (Pradhan et al., 2017; Sood et al., 2018). Ashwagandha is also purported to influence HPA axis activity (Lopresti et al., 2022). Therefore, changes in sex hormones, blood glucose concentrations, thyroid activity, and the sympathetic response were examined over time. Overweight or mildly obese men and women, with self-reported stress and fatigue, were recruited as obesity is associated with an increased risk of reduced testosterone concentrations in men (Kelly and Jones, 2015), sex hormone dysfunction in women (Gambineri and Pelusi, 2019; Naderpoor et al., 2015), increased sympathetic response (Lemche et al., 2016), blood glucose dysregulation (Martyn et al., 2008), increased oxidative stress (Tobore, 2020), and thyroid dysfunction (Sanyal and Raychaudhuri, 2016). It was hypothesized that ashwagandha supplementation would be associated with reduced self-reported stress and fatigue levels, and its mechanisms of action may be due to its influence on endocrine hormone activity, oxidative stress, and/or the sympathetic response.

## Materials and methods

### Study design

**Figure 1.**
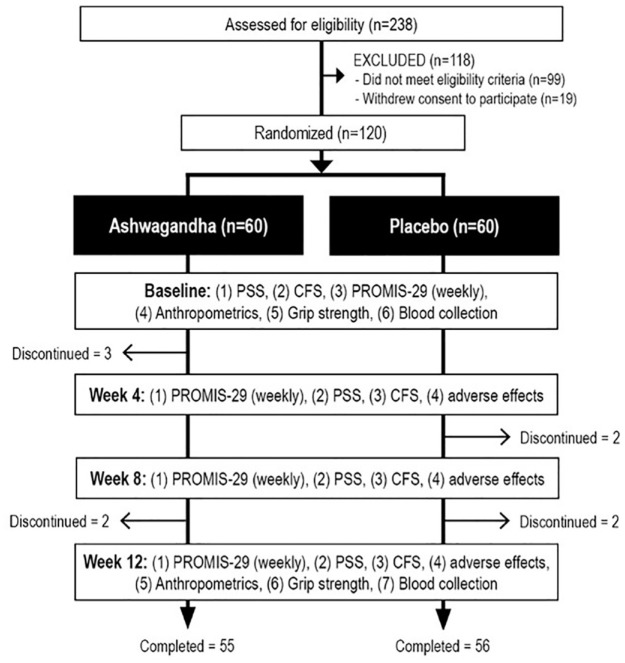
Study design. CFS: Chalder Fatigue Scale; PROMIS-29: Patient-Reported Outcomes Measurements Information System—29; PSS: Perceived Stress Scale.

This was a two-arm, parallel-group, 12-week, single-center, randomized, double-blind, placebo-controlled trial (Figure 1). The trial protocol was approved by the Human Research Ethics Committee at the National Institute of Integrative Medicine (approval number 0092E_2021), and all participants gave informed consent electronically. This study was prospectively registered with the Australian and New Zealand Clinical Trials Registry (Trial Registration number: ACTRN12621001551886).

### Sample size calculation

Previous studies investigating ashwagandha’s effects on stress and anxiety, with both men and women, and using a similar dosage to the current study, had sample sizes ranging from 60 to 80 participants, with effect sizes ranging from 0.63 to 0.74 (Abedon et al., 2008; Chandrasekhar et al., 2012; Langade et al., 2019; Langade et al., 2021; Lopresti et al., 2019a; Salve et al., 2019). We predicted a more conservative effect size of 0.5, thereby requiring a sample size of 51 per group. This gave an 80% chance of finding an effect at a statistical significance of 0.05. In this study, 60 participants per group were recruited (120 participants in total). Based on the 120 people recruited, there was suitable power to find an effect, even with an approximate attrition rate of 10%.

#### Recruitment

Participants were recruited via social media advertisements and an email database between March and June 2022. Interested volunteers were directed to a website that provided details about the trial and a link to complete an online screening and application form. Before completing this application, volunteers were required to agree to provide such background information; however, an informed consent form was not completed until after the telephone interview, where applicants eligibility to participate in the study was further assessed. The application form included questions to obtain relevant background details, health history, medication use, supplement use, drug and alcohol use, and questionnaires comprising the Patient-Reported Outcome Measurement Information System-29 (PROMIS-29)—Fatigue Subscale and the four-item Patient Health Questionnaire (PHQ-4), which is a reliable and valid brief self-report measure to detect anxiety and depressive disorders in adults (Kroenke et al., 2009). To provide an indication of menopausal status, women were also asked if they had experienced a menstrual episode in the past 12 months; a negative response suggested they were post-menopausal.

### Participants

Interested volunteers, evaluated as likely eligible, were contacted via telephone for further assessment of their eligibility and to obtain demographic details. Participants meeting the eligibility criteria were then required to complete an online informed consent form and to have a sample of their non-fasted blood collected from a local pathology collection center. This blood sample was used to measure glycated hemoglobin (HbA1C), liver function, full blood count (FBC), renal function, and thyroid-stimulating hormone (TSH). Participants with results significantly outside the reference ranges were ineligible to participate in the study. Participant eligibility criteria are outlined below:

#### Inclusion criteria

Healthy males and females aged between 40 and 75 years; experiencing low energy; fatigue, based on a score of 9 or more on the PROMIS-29 fatigue subscale score; and moderate-to-high stress, based on a score of 14 or higher on the Perceived Stress Scale (PSS) (primary outcome); a non-smoker; with a body mass index (BMI) between 25 and 35 kg/m^2^; reporting no plan to change their diet or start new treatments during the study period, and willing to comply with all study procedures.

#### Exclusion criteria

Interested volunteers were considered ineligible if they anticipated experiencing a major stressor or life event during the study that may affect their psychological or physical status; if they suffered from a newly diagnosed or unstable medical condition including, but not limited to, diabetes, hyper- or hypotension, cardiovascular disease, a gastrointestinal disease requiring regular use of medication, gallbladder disease/gallstones/biliary disease, autoimmune disease, endocrine disease, renal disease, hepatic disease, genital anatomical deformities, abnormal secondary sexual characteristics, spinal cord injury, benign prostatic hypertrophy, acute genitourinary disorder or history of genital surgery, acute or chronic pain conditions, or cancer or malignancy. Moreover, participants were ineligible if they were diagnosed with a serious neurological or psychiatric disorder, diagnosed with erectile dysfunction, or diagnosed with any physical disability that may limit sexual function. Regularly using nutritional supplements, vitamins, or medications that may influence study measures were also exclusion criteria. Any medication changes in the last 3 months or an expectation of changing medication use during the study also resulted in exclusion from the study. People who reported a current or 12-month history of illicit drug abuse or alcohol intake greater than 14 standard drinks per week were also ineligible to participate in the study. Women who were pregnant, breastfeeding, or intending to fall pregnant were also ineligible. People who experienced significant injuries over the previous 12 months; were hypersensitive to herbs, spices, or dietary supplements; reported any planned major lifestyle change during the study period; or had participated in any other clinical trial in the previous 3 months were also ineligible to participate in the study.

#### Procedures

All consenting and eligible volunteers were required to attend the office of Clinical Research Australia on two occasions, at the commencement and conclusion of his or her 12-week participation in the study. Prior to attending each visit, participants were required to visit his or her local pathology collection center and provide a blood sample. The blood sample tested for liver function, kidney function, complete blood count, TSH, and HbA1C. Prior to the initial visit, volunteers with abnormal results were informed that they were ineligible to participate. Each assessment lasted approximately 60 min and involved anthropometric measurements, a blood collection, measurement of Heart Rate Variability (HRV), a grip strength test, and completion of questionnaires. Participants were given a 12-week supply of capsules. Participants took 1 capsule (containing either 200 mg of an ashwagandha extract or a matching placebo) twice daily, either with or without food, for 12 weeks. Participants were also required to complete a weekly questionnaire that asked about any adverse and/or significant events.

### Intervention

The intervention, containing a standardized ashwagandha (*Withania somnifera)* root extract, Witholytin®, was supplied by Verdure Sciences Inc. Ashwagandha and placebo capsules were identical in appearance, being matched for color, shape, and size. Each capsule of the active intervention contained 200 mg hydroalcoholic extract of the roots of ashwagandha (*Withania somnifera*) standardized to 1.5% total withanolides by high-performance liquid chromatography using United States Pharmacopeia Methodology. The same excipient, cellulose, was used in the preparation of both the ashwagandha and placebo capsules. Participants were instructed to maintain their existing lifestyle as closely as possible and take 1 capsule, twice daily (morning and evening), with or without food, for 12 weeks, delivering 400 mg of Witholytin® daily. Capsule adherence was assessed by the twice daily use of a mobile phone pill monitoring/reminder application, by weekly questionnaires asking participants to provide an estimate of the consistency of capsule intake (0–100%), and by the return of unused capsules at the final assessment. Treatment blinding was evaluated by asking participants to predict their group allocation (ashwagandha, placebo, or unsure) at the end of the study.

#### Randomization

Consenting and eligible participants were randomly allocated to one of two groups (ashwagandha or placebo). A randomization calculator was used to ensure sequence concealment and to create a randomization structure comprising 12 randomly permuted blocks, containing 10 participants per block, with a 1-to-1 ratio. Study identification numbers were allocated to participants based on attendance at their first in-person visit. All capsules were packaged in matching bottles labeled with two intervention codes. The sponsor held the identity of the intervention codes until all data were analyzed. Investigators and study participants were blind to the treatment group allocation until all outcome data were collected.

### Outcome measures

#### Primary outcome measure

##### PSS total score

The PSS is a widely used self-report questionnaire measuring the degree to which situations in a person’s life are considered stressful (Ezzati et al., 2014). It is a validated 10-item stress perception scale (Ribeiro Santiago et al., 2020). Items are designed to identify how unpredictable, uncontrollable, and overloaded a person finds their life. The PSS also includes questions about a person’s current level of stress. The questions in the PSS ask about feelings and thoughts during the previous 4 weeks. Respondents provided ratings on a 5-point scale (never to very often), with items associated with being upset unexpectantly, control, stress/nervousness, ability to handle personal problems, coping, life irritations, and anger options. Scores on the PSS range from 0 to 40 with higher scores indicating higher perceived stress. Scores of 0 to 13 are considered low stress, 14 to 26 moderate stress, and 27 to 40 high perceived stress. The PSS was completed every 4 weeks. PSS scores are also positively associated with cortisol concentrations (Walvekar et al., 2015).

#### Secondary Outcome Measures

##### Chalder Fatigue Scale (CFS)

The CFS is a validated 11-item scale for assessing symptoms of mental and physical fatigue (Jing et al., 2016). Respondents rated each item on a 4-point scale (e.g., “less than usual” to “much more than usual” or “better than usual” to “much worse than usual”), with items associated with tiredness, rest, feeling sleepy, motivation, energy, muscular strength, weakness, concentration, and speech. The CFS was completed every 4 weeks.

##### PROMIS-29 + Interest in Sexual Activity subscale score

The PROMIS-29 is a validated, health-related, quality-of-life self-report questionnaire (Kroenke et al., 2018). As a primary investigation, scores were calculated for physical, mental, and interest in sexual activity. As an exploratory analysis, subscale scores for physical function, anxiety, depression, fatigue, sleep, social function, pain interference, and pain intensity were examined. There are four questions associated with each subscale (except interest in sexual activity (two questions) and pain intensity (one rating scale)). The participants completed the PROMIS-29 weekly and responded based on the past 7 days.

##### Sex hormone concentrations, malondialdehyde (MDA) concentrations, and fasting glucose

Blood samples for plasma concentrations of total testosterone (TT), free testosterone (FT), estradiol, LH, DHEA-S, MDA, and fasting glucose were collected at baseline and week 12. Blood samples were collected in the morning between 8:00 am and 12:00 pm, in a fasted, non-exercised state, with no alcohol being consumed the evening before providing the sample. Details about sample collections and analyses and normative data on sex hormones are included in the Supplemental file.

##### Glycated hemoglobin (HbA1c) and thyroid-stimulating hormone (TSH)

HbA1c and TSH concentrations were taken during the baseline and week-12 assessments. The Roche Cobas c513 analyzer and the Roche TINA-quant Hemoglobin A1c Gen.3 Whole Blood assay were used.

##### Heart Rate Variability (HRV)

Five-minute HRV measurements were taken during the baseline and week-12 assessments. Participants were instructed to remain seated and breathe normally for 5 min for the duration of the HRV measurement. The Kubios HRV scientific software program (Version 4.0.2), Kubios Oy, and Polar H10 heart rate sensor were used at a sampling frequency of 100 Hz. For the Kubios software, automatic beat correction was set at medium with a 5% acceptance threshold, the RR time series interpolation rate was 4 Hz, and the RR interval detrending method was set at smoothness priors to remove very low-frequency trend components with a smoothness parameter of 500. The root mean square of successive differences (RMSSD) measurement was calculated since it is one of the most commonly reported short-term measures of HRV (Shaffer et al., 2014). The RMSSD refers to the beat-to-beat variance in heart rate and is the primary time-domain measure used to estimate the vagally mediated changes reflected in the heart rate (Shaffer et al., 2014). RMSSD is more influenced by the parasympathetic nervous system and its statistical robustness makes it well-suited for short-term time windows (Pham et al., 2021; Shaffer and Ginsberg, 2017; Shaffer et al., 2014).

##### Grip strength

Grip strength is a good indicator of overall body strength (Bohannon, 2019). Therefore, measurements were taken during the baseline and week-12 assessments. A Jamar Plus+ Digital Hand Dynamometer was used. Participants were instructed to hold the dynamometer in one hand while in a seated position with forearms resting on the armrests of the chair. The participants were required to squeeze the dynamometer as hard as possible for 5–10 s followed by a 5–10-s rest period. This was repeated using the same hand before changing to the other hand and repeating the procedure. Grip strength was measured in kilograms of pressure, with the highest score for each hand recorded.

##### Anthropometric measures

As a safety measure, several anthropometric measurements were taken over time. These included BMI, waist circumference (WC), and waist-to-hip ratio (WHR). WC was measured using a plastic tape measure at the narrowest point of the waist and the hip circumference was measured at the widest point of the hips when viewed from a lateral perspective.

##### Liver function, renal function, and full blood count (FBC)

As a safety measure, a blood sample was collected at baseline and week 12 to identify changes in liver function, renal function, and FBC. Blood samples were collected at any time of the day with no fasting requirements.

##### Adverse effects

To assess the tolerability of capsule intake, participants completed online questionnaires every 7 days asking for adverse effects, medication/ supplement changes, and illnesses/injuries experienced over the previous week.

### Statistical analysis

Outcome analyses were conducted using intention-to-treat principles, with all participants retained in his or her originally assigned groups. Generalized linear mixed models (GLMMs) assessed for differences between intervention groups on primary and secondary outcomes over time, with intervention effects assessed via entry of intervention group (placebo and ashwagandha) × time interaction. The time points examined for each outcome included: (1) PSS and CFS total score (weeks 0, 4, 8, and 12); (2) PROMIS-29, comprising physical, mental, and sexual health scores (baseline, and the means of weeks 1–4, 5–8, and 9–12); and (3) TT, FT, estradiol, LH, DHEA-S, fasting glucose, HbA1c, MDA, BMI, WC, WHR, grip strength, and HRV (RMSSD) (weeks 0 and 12). Due to significant sex differences in sex hormone concentrations and body compositions, separate analyses were conducted by sex. Random intercepts were utilized in each GLMM, and the covariates of age and BMI were included as fixed effects. Where applicable, gamma (with log link function) and normal (with identity link function) target distributions were used. Appropriate covariance structures with the best model fit were used to model correlation associated with repeated time measurements in gamma and linear models. Robust estimations were used to handle any violations of model assumptions, and least significance tests were used for within-group time contrasts. Intervention group differences at time points were assessed using simple contrasts. All data were analyzed using SPSS (version 26; IBM, Armonk, NY, USA).

## Results

### Study population

As detailed in Figure 1, there were 238 people who completed the online screening questionnaire, with 99 people not meeting the eligibility criteria and 43 people withdrawing consent before participation in the study. During the study, five participants allocated to the ashwagandha group and four participants from the placebo group withdrew from the study. Fifty-five participants from the ashwagandha group and 56 participants from the placebo group were used for analysis. Participants from the ashwagandha groups who withdrew from the study provided the following reasons: personal issues (*n* = 1), sore ankle (*n* = 1), reduced mood and tiredness (*n* = 1), and no reason given (*n* = 2). Participants from the placebo groups who withdrew from the study provided the following reasons: complications after COVID (*n* = 1), not experiencing improvements (*n* = 1), COVID infection (*n* = 1), and itching on hands and rashes on the body (*n* = 1). Participant baseline sociodemographic and clinical characteristics are detailed in Table 1.

**Table 1.** Baseline sociodemographic and clinical characteristics.

	Ashwagandha (*n* = 60)	Placebo (*n* = 60)	*p*-Value
Age			
Mean	53.72	55.45	0.307^a^
SE	1.20	1.19	
BMI			
Mean	30.00	29.45	0.382^a^
SE	0.44	0.45	
Marital status			
Single (*n*)	23	16	0.242^b^
Married/de facto (*n*)	37	44	
Education			
Secondary (*n*)	33	28	0.439^b^
Tertiary (*n*)	12	18	
Post-graduate (*n*)	15	14	
Blood pressure—systolic (mmHg)			
Mean	133.85	132.08	0.493^a^
SE	1.72	1.91	
Blood pressure—diastolic (mmHg)			
Mean	86.73	83.83	0.104^a^
SE	1.43	1.05	
International Physical Activity Questionnaire (IPAQ) category			
Low (*n*)	29	33	0.640^b^
Moderate (*n*)	28	51	
High (*n*)	3	7	
WHR			
Mean	0.90	0.91	0.549^a^
SE	0.01	0.01	
Mean grip strength (L&R) (kg of pressure)			
Mean	37.61	38.14	0.827^a^
SE	1.76	1.71	
PSS			
Mean	19.82	19.63	0.816^a^
SE	0.55	0.57	
CFS			
Mean	16.82	16.85	0.968^a^
SE	0.59	0.57	
PROMIS-physical			
Mean	42.94	43.46	0.674^a^
SE	0.84	0.93	
PROMIS-mental			
Mean	40.76	41.28	0.615^a^
SE	0.71	0.73	
HRV (RMSSD)			
Mean	27.05	31.38	0.286^a^
SE	2.80	2.90	
Glucose (mmols/L)			
Mean	5.42	5.68	0.024^a^
SE	0.06	0.10	
HbA1C (%)			
Mean	140.30	142.00	0.424^a^
SE	1.60	1.39	
TSH (mIU/L)			
Mean	1.87	1.54	0.039^a^
SE	0.12	0.10	

a Independent-samples *t*-test.

b Pearson chi-square test.

### Outcome measures

#### Primary outcome measure

##### PSS total score

As demonstrated in Table 2, based on the GLMM, there was no statistically significant time × group interaction in PSS total score over time (*F*_3,439_ = 0.24, *p* = 0.867). In the ashwagandha group, there was a statistically significant 38.59% improvement (reduction) in PSS total score from baseline to week 12 (*T*(439) = 9.39, *p* < 0.001) and a statistically significant 35.63% improvement (reduction) in the placebo group (*T*(439) = 10.47, *p* < 0.001).

**Table 2.** Change in PSS and CFS (all participants; estimated marginal means).

	Ashwagandha (*n* = 60)					Placebo (*n* = 60)					*p*-Value^b^	Percentage change: Week 0 to week 12	
	Week 0	Week 4	Week 8	Week 12	*p*-Value^a^	Week 0	Week 4	Week 8	Week 12	*p*-Value^a^		Ashwagandha	Placebo
PSS (total score)													
Mean	19.80	15.35	14.00	12.16	<0.001	19.73	15.16	14.41	12.70	<0.001	0.867	−38.59	−35.63
SE	0.56	0.82	0.81	0.85		0.56	0.75	0.73	0.74				
CFS (total score)													
Mean	16.81	11.72	11.25	9.11	<0.001	16.85	11.43	12.49	11.55	<0.001	0.016	−45.81	−31.45
SE	0.58	0.74	0.73	0.65		0.56	0.55	0.73	0.76				

Results (estimated means) are generated from generalized mixed-effects models adjusted for age and BMI.

a *p*-Values are generated from repeated measures generalized mixed-effects models adjusted for age and BMI (time effects baseline, week 4, 8, and 12).

b *p*-Values are generated from repeated measures generalized mixed-effects models adjusted for age and BMI (time × group interaction).

#### Secondary outcome measures

##### Chalder Fatigue Scale

Using a GLMM, based on data from the total sample, there was a statistically significant time × group interaction in CFS over time (*F*_3,439_ = 3.49, *p* = 0.016) (Table 2). In the ashwagandha group, there was a statistically significant improvement (reduction) (45.81%) in the CFS total score from baseline to week 12 (*T*(439) = 9.81, *p* < 0.001) and a statistically significant improvement (reduction) (31.45%) in the placebo group (*T*(439) = 6.92, *p* < 0.001).

##### PROMIS-29 + Interest in Sexual Activity subscale score

There was no statistically significant time × group interaction in the PROMIS physical (*F*_3,459_ = 0.56, *p* = 0.642), mental (*F*_3,459_ = 0.63, *p* = 0.626), or sexual interest scores (*F*_3,459_ = 0.41, *p* = 0.747) over time, based on the GLMM (Table 3).

**Table 3.** Change in PROMIS-29 subscale scores (all participants; estimated marginal means).

	Ashwagandha (*n* = 60)					Placebo (*n* = 60)					p-Value^b^	Percentage change: Week 0 to week 12	
	Week 0	Weeks 1–4 (mean)	Weeks 5–8 (mean)	Weeks 9–12 (mean)	*p*-Value^a^	Week 0	Weeks 1–4 (mean)	Weeks 5–8 (mean)	Weeks 9–12 (mean)	*p*-Value^a^		Ashwagandha	Placebo
PROMIS-29 (Physical Score)													
Mean	42.78	44.71	45.60	47.66	<0.001	43.51	44.28	45.30	47.02	<0.001	0.642	11.41	8.07
SE	0.80	0.83	0.76	0.76		0.88	0.88	0.87	0.81				
PROMIS-29 (Mental Score)													
Mean	40.84	36.15	33.10	30.10	<0.001	41.22	36.90	34.02	31.49	<0.001	0.626	−26.30	−23.61
SE	0.71	0.87	0.87	0.90		0.72	0.71	0.74	0.75				
PROMIS-29 (Sexual Interest)													
Mean	47.55	45.92	45.59	46.03	0.091	45.88	44.58	44.91	45.50	0.656	0.747	−3.20	−0.83
SE	0.88	0.61	0.80	0.86		0.84	0.70	0.78	0.82				

Results (estimated means) are generated from generalized mixed-effects models adjusted for age and BMI.

a *p*-Values are generated from repeated measures generalized mixed-effects models adjusted for age and BMI (time effects baseline, and the means of weeks 1–4, 5–8, and 9–12).

b *p*-Values are generated from repeated measures generalized mixed-effects models adjusted for age and BMI (time × group interaction).

##### Blood markers and hormones

As detailed in Table 4, for men, there was no statistically significant time × group interactions in blood concentrations except for FT (*F*_1,104_ = 4.00, *p* = 0.048) and LH (*F*_1,103_ = 6.06, *p* = 0.016). In the ashwagandha group, there was a statistically significant 12.87% increase in FT (*T*(104) = 1.11, *p* = 0.002) and a non-significant decline of 1.69% in the placebo group (*T*(104) = 0.29, *p* = 0.772) from baseline to week 12. In the placebo group, there was a statistically significant 11.13% decrease in LH over time (*T*(103) = 2.14, *p* = 0.034) and a non-significant increase of 7.15% in the ashwagandha group (*T*(103) = 1.25, *p* = 0.213). Although not reaching statistical significance, there was a trend suggesting a between-group difference in change in MDA concentrations over time (*F*_1,106_ = 3.70, *p* = 0.057). In the ashwagandha group, there was a statistically significant increase (84.69%) in MDA concentrations from baseline to week 12 (*T*(106) = 2.91, *p* = 0.004) and a non-significant increase of 5.45% in the placebo group (*T*(106) = 0.24, *p* = 0.813).

**Table 4.** Change in blood markers and hormones (men only; estimated marginal means).

	Ashwagandha (*n* = 28)			Placebo (*n* = 28)			*p*-Value^b^	Percentage change: Week 0 to week 12	
	Week 0	Week 12	*p*-Value^a^	Week 0	Week 12	*p*-Value^a^		Ashwagandha	Placebo
Fasting glucose (mmol/L)									
Mean	5.53	5.59	0.443	5.91	5.96	0.474	0.901	1.08	0.86
SE	0.11	0.12		0.11	0.13				
Glycated hemoglobin (%)									
Mean	5.42	5.36	0.045	5.65	5.66	0.733	0.137	−1.13	0.27
SE	0.06	0.05		0.04	0.06				
Malondialdehyde (ng/mL)									
Mean	38.35	70.82	0.004	34.95	36.86	0.813	0.057	84.69	5.45
SE	7.68	13.29		6.41	5.98				
TT (ng/mL)									
Mean	4.53	4.86	0.140	4.33	4.43	0.538	0.424	7.22	2.26
SE	0.28	0.28		0.16	0.16				
FT (pg/mL)									
Mean	8.64	9.75	0.002	10.24	10.07	0.772	0.048	12.87	−1.69
SE	0.66	0.76		0.73	0.62				
Oestradiol (pg/mL)									
Mean	66.08	84.28	0.023	52.12	67.26	0.106	0.948	27.54	29.03
SE	9.86	13.04		6.70	9.78				
Dehydroepiandrosterone sulfate (µg/mL)									
Mean	1.19	1.31	<0.001	1.11	1.27	0.001	0.367	9.91	14.63
SE	0.12	0.14		0.07	0.07				
TSH (mIU/L)									
Mean	2.43	2.22	0.134	1.80	1.81	0.857	0.179	−8.64	0.56
SE	0.35	0.31		0.22	0.22				

Results (estimated means) are generated from generalized mixed-effects models adjusted for age and BMI.

a *p*-Values are generated from repeated measures generalized mixed-effects models adjusted for age and BMI (time effects baseline and week 12).

b *p*-Values are generated from repeated measures generalized mixed-effects models adjusted for age and BMI (time × group interaction).

As detailed in Table 5, for women, there were no statistically significant time × group interactions in blood concentrations. However, there was a trend suggesting a between-group difference in change in estradiol concentrations over time (*F*_1,95_ = 2.25, *p* = 0.137). In the ashwagandha group, there was a statistically significant increase (59.72%) in estradiol concentrations from baseline to week 12 (*T*(95) = 2.22, *p* = 0.029) and a non-significant reduction of 1.49% in the placebo group (*T*(95) = 0.06, *p* = 0.955).

**Table 5.** Change in blood markers and hormones (women only; estimated marginal means).

	Ashwagandha (*n* = 24)			Placebo (*n* = 28)			*p*-Value^b^	Percentage change: Week 0 to week 12	
	Week 0	Week 12	*p*-Value^a^	Week 0	Week 12	*p*-Value^a^		Ashwagandha	Placebo
Fasting glucose (mmols/L)									
Mean	5.24	5.29	0.170	5.29	5.23	0.556	0.197	0.95	−1.13
SE	0.09	0.08		0.11	0.09				
Glycated hemoglobin (%)									
Mean	5.45	5.43	0.434	5.43	5.42	0.589	0.887	−0.37	−0.18
SE	0.04	0.04		0.04	0.04				
Malondialdehyde (ng/mL)									
Mean	68.95	78.3	0.472	38.58	55.27	0.098	0.429	13.56	43.26
SE	19.35	17.5		10.74	11.39				
TT (ng/mL)									
Mean	0.65	0.73	0.036	0.6	0.66	0.038	0.865	12.31	10.00
SE	0.05	0.06		0.05	0.05				
FT (pg/mL)									
Mean	2.23	2.16	0.703	1.68	1.53	0.190	0.560	−3.14	−8.93
SE	0.37	0.36		0.31	0.3				
Oestradiol (pg/mL)									
Mean	90.94	145.25	0.029	89.27	87.94	0.955	0.137	59.72	−1.49
SE	14.92	27.36		19.57	15.46				
Luteinising hormone (mIU/mL)									
Mean	40.63	48.94	0.168	48.14	48.83	0.832	0.253	20.45	1.43
SE	4.68	5.29		5.26	5.74				
Dehydroepiandrosterone sulfate (µg/mL)									
Mean	1.13	1.15	0.749	1.11	1.05	0.109	0.319	1.77	−5.41
SE	0.12	0.15		0.1	0.1				
TSH (mIU/L)									
Mean	1.98	2.01	0.806	1.68	1.82	0.504	0.632	1.52	8.33
SE	0.18	0.2		0.17	0.28				

Results (estimated means) are generated from generalized mixed-effects models adjusted for age and BMI.

a *p*-Values are generated from repeated measures generalized mixed-effects models adjusted for age and BMI (time effects baseline and week 12).

b *p*-Values are generated from repeated measures generalized mixed-effects models adjusted for age and BMI (time × group interaction).

##### Heart rate variability

For the total sample, there was a statistically significant time × group interaction in RMSSD over time (*F*_1,206_ = 8.86, *p* = 0.003), based on the GLMM (Table 6). In the ashwagandha group, there was a non-significant increase (9.08%) in RMSSD from baseline to week 12 (*T*(206) = 1.48, *p* = 0.141) and a statistically significant reduction (−18.84%) in the placebo group (*T*(206) = 2.38, *p* = 0.018).

**Table 6.** Change in HRV (estimated marginal means).

	Ashwagandha (*n* = 57)			Placebo (*n* = 58)			*p*-Value^b^	Percentage change: Week 0 to week 12	
	Week 0	Week 12	*p*-Value^a^	Week 0	Week 12	*p*-Value^a^		Ashwagandha	Placebo
RMSSD									
Mean	26.44	28.84	0.141	31.37	25.46	0.018	0.003	9.08	−18.84
SE	2.425	2.6		3.04	1.65				

Results (estimated means) are generated from generalized mixed-effects models adjusted for age and BMI.

a *p*-Values are generated from repeated measures generalized mixed-effects models adjusted for age and BMI (time effects baseline and week 12).

b *p*-Values are generated from repeated measures generalized mixed-effects models adjusted for age and BMI (time × group interaction).

##### Grip strength measures

For both men and women (Supplemental Tables 2 and 3), there were no statistically significant time × group interactions in grip strength over time, based on the GLMM.

### Measurement of treatment compliance

Capsule bottles with remaining capsules were returned on the week-12 assessment, and participants completed a daily medication monitoring phone application. Based on these details, 95% of participants who completed the study took over 80% of their capsules.

### Efficacy of participant blinding

To assess the effectiveness of participant blinding, at the end of the study, participants were asked to predict their condition allocation (i.e., ashwagandha, placebo, unsure). Concealment of group allocation was considered high, with 64% of participants being unsure of group allocation or guessing incorrectly.

### Self-reported adverse reactions and safety measures

As detailed in Table 7, no serious adverse reactions were reported by participants, and a similar frequency of adverse reactions was reported in both groups. Three participants withdrew from the study due to low or moderate severity of self-reported adverse reactions believed to be associated with capsule intake. In the ashwagandha group, one participant withdrew due to self-reported ankle pain and one due to reduced mood/tiredness. In the placebo group, one participant withdrew due to self-reported skin itchiness/rashes. There were no reports of any adverse reactions in 78% of participants in the ashwagandha group and 75% in the placebo group. An analysis of changes in blood safety measures from baseline to week 12 demonstrated there were no statistically significant between-group differences in changes in liver function, renal function, or complete blood count over time (Supplemental Table 4). For both men and women (Supplemental Tables 2 and 3), there were no statistically significant time × group interactions in BMI, WC, and WHR, based on the GLMM.

**Table 7.** Self-reported adverse reactions.

	Placebo (%)	Ashwagandha (%)
Any adverse reaction	15 (25)	13 (22)
Any serious adverse reaction	0 (0)	0 (0)
Any adverse reaction leading to study discontinuation	1 (2)	2 (3)
Adverse reactions that occurred in > 1 participant		
Digestive disturbances	10 (17)	6 (10)
Mood disturbances/changes	4 (7)	4 (7)
Headaches/migraines	2 (3)	1 (2)
Increased appetite	2 (3)	1 (2)
Itchy skin	2 (3)	0 (0)
Increased tiredness	1 (2)	1 (2)

## Discussion

In this 12-week, randomized, double-blind, placebo-controlled trial on overweight or mildly obese men and women aged 40–75 years, supplementing with 200 mg of an ashwagandha root extract (Witholytin®) twice daily was associated with a significant reduction in their stress levels based on the PSS (primary outcome measure); however, the improvements were not significantly different to participants taking a placebo. Based on the CFS, there was a significant reduction in self-reported fatigue symptoms in the ashwagandha group compared to the placebo group, and participants taking ashwagandha also experienced a significant increase in HRV, as measured by RMSSD, compared to the placebo group. However, there were no significant between-group differences in other self-report outcome measures. In the men taking ashwagandha, there was a significant increase in the blood concentrations of FT and LH compared to the placebo group, and a trend suggesting a greater increase in MDA concentrations. Moreover, there was a trend of a greater increase in estradiol concentrations in women taking ashwagandha. However, there were no significant between-group differences in changes in DHEA-S, TT, HbA1c, fasting glucose, and grip strength. Ashwagandha intake was well-tolerated, with both groups experiencing a similar number of self-reported adverse effects. Further evidence of the safety of ashwagandha supplementation over 12 weeks is demonstrated by findings of no significant changes in blood safety measures comprising full blood count, liver function, and renal function, and anthropometric measurements comprising BMI, waist and hip circumference.

In this study, there were no significant between-group differences in changes in the PSS, the primary outcome measure and a validated self-report measure of perceived stress in adults. However, this finding contrasts with other studies investigating the effects of ashwagandha using the PSS, which have found positive (stress-reducing) effects on PSS scores over time (Chandrasekhar et al., 2012; Salve et al., 2019). In this study, there was a significant improvement (reduction) in the PSS score, with the ashwagandha group improving by 39.5%, but there was also a significant placebo effect, whereby PSS scores were reduced by 35.2%. Surprisingly, significant placebo effects have not typically occurred in previous studies conducted on ashwagandha, which may account for the non-significant finding in this study. However, in this study, the magnitude of change in PSS scores in the ashwagandha group was similar to those identified in previous studies. For example, in a 60-day study on Indian adults with high stress, PSS scores reduced by 44% in participants taking an ashwagandha extract for 8 weeks, but there was only a 5.5% reduction in the PSS scores in the placebo groups (Chandrasekhar et al., 2012). In another 8-week study on healthy, stressed Indian adults, the administration of an ashwagandha extract reduced the PSS scores by 33%, but PSS scores only reduced by 3.6% in the placebo group (Gopukumar et al., 2021). Reasons for these dramatically different placebo effects require future investigation, although differences in the populations recruited could be a factor accounting for this finding. Moreover, it has been identified in some studies that cultural factors and beliefs may affect the placebo response. In a review of placebo treatments for ulcers, there was significant variation in the placebo response across countries, with German participants experiencing a 59% placebo response compared to only 7% in Brazilian participants (Moerman, 2000). As ashwagandha use is more commonplace in India, this may also account for the lack of placebo response in studies conducted in India.

Despite no significant difference between the groups in the change of PSS scores (primary outcome), there was a statistically significant between-group difference in change in HRV over time. However, this finding needs to be tentatively considered since there was a statistically significant 18.8% reduction in HRV in the placebo group, and only a non-significant increased trend of 9.1% in the ashwagandha group. HRV is the measurement of time between successive beats of the heart and provides a measure of the interplay between the parasympathetic and sympathetic nervous systems. HRV is inversely associated with cortisol concentrations (Mishica et al., 2021) and is positively correlated with low testosterone concentrations in men (Ermis et al., 2010). The between-group differences in HRV suggest that ashwagandha supplementation was associated with increased parasympathetic activity, which is typically associated with reduced stress levels. However, this physiological change from ashwagandha supplementation did not lead to reduced self-reported perceived stress as measured by the PSS. A reason for this contrasting finding may be because the change in HRV was not of significant magnitude to result in noticeable reductions in perceived stress for individuals. Moreover, differences in testing protocols may account for these divergent findings as the PSS is based on self-reports of stress-related changes over the previous 4 weeks, and in this study, HRV was a physiological single-point, 5-min measure. It has been reported that ashwagandha has HPA axis-modulating effects so further investigations into the effects of ashwagandha on HRV may be useful (Lopresti et al., 2022; Speers et al., 2021). Interestingly, in this study, a post-hoc exploratory analysis did not reveal any significant relationship between changes in HRV and other outcome measures collected in this study.

A significant reduction in fatigue symptoms in the ashwagandha group compared to the placebo group, as measured by the CFS, was identified in the present study. The positive result from the CFS is supported by a meta-analysis examining the effects of ashwagandha on physical performance, which included fatigue symptoms. The results of the review from 13 studies and 615 adults concluded that the pooled effect of ashwagandha administration was large (effect size of 1.18) and that future studies demonstrating positive effects in terms of fatigue reduction were likely to be meaningful (Bonilla et al., 2021). It could be theorized that the decrease in fatigue symptoms may result from increases in sex hormones in both men (FT) and women (estradiol) since research indicates that a relationship may exist between sex hormone concentrations and fatigue (Chahal and Drake, 2007; Mani and Clavijo, 2022; Moller et al., 2013; Petering and Brooks, 2017). However, an exploratory analysis revealed that changes in these sex hormones were not significantly correlated with changes in the CFS score. This suggests that other mechanisms associated with reduced fatigue levels may be at play. These mechanisms require investigation in future trials and could be due to their influence on the HPA axis (Lopresti et al., 2019b), antioxidant activity (Alam et al., 2011), and/or anti-inflammatory effects (Chandra et al., 2012).

Despite ashwagandha being associated with increases in FT and estradiol concentrations over time in men and women, respectively, concentrations in these sex hormones remained within normal ranges. It is important to note that in men, estradiol concentrations increased by 28% and 29% in the ashwagandha and placebo groups, respectively. Despite these similar changes, at week 12, estradiol concentrations in men taking ashwagandha were slightly above normal ranges. Similar increases in estradiol occurred in both the placebo and ashwagandha groups, with baseline estradiol concentrations being slightly higher in the ashwagandha group. Moreover, since BMI and obesity in men are associated with increased estradiol concentrations and aromatase activity (Cohen, 2008; Colleluori et al., 2020), these changes are considered not clinically meaningful. However, this observation should be monitored in further studies on ashwagandha, as plant ingredients have been shown in some trials to have estrogenic effects in males (Henley et al., 2007; Santti et al., 1998).

A further observation in men was a trend suggesting a between-group difference in changes in MDA concentrations. This was signified by an 85% increase in MDA concentrations from baseline to week 12 in the ashwagandha group and only a 5% increase in the placebo group. As there was substantial variability in MDA concentrations over time in the ashwagandha group at week 12, this finding should be interpreted cautiously. In a previous ashwagandha study, MDA concentrations were reduced by 18% following 6 months of supplementation in healthy adults (Kuchewar et al., 2014). In a review by Khoubnasabjafari and colleagues (2015), it was concluded that due to technical problems and inconsistent findings in healthy people and some psychiatric disorders, the reliability of MDA as a biomarker of oxidative stress requires re-evaluation. Despite this, it remains important to continue to investigate if ashwagandha supplementation has pro-oxidant effects in clinical and non-clinical populations.

In women, there was no statistically significant between-group difference in estradiol concentrations; however, the ashwagandha group did experience a significant 59% increase in estradiol concentrations from baseline to week 12. Even though the sample sizes were too small to formulate robust conclusions, an exploratory analysis of changes in estradiol concentrations in perimenopausal women taking ashwagandha (*n* = 10) revealed an increase of 137% in estradiol concentrations from baseline to week 12, compared to a smaller 26% increase in post-menopausal women (*n* = 14). In contrast, in the placebo group, estradiol concentrations increased by 6% in perimenopausal women (*n* = 12) and reduced by 14% in post-menopausal women (*n* = 16). However, it is important to note that despite an increase in estradiol in the ashwagandha group, concentrations continued to remain within normal ranges. Further research on the effects of ashwagandha on sex hormone concentrations in women of varying ages and menstrual status requires further investigation. In a randomized controlled trial on perimenopausal women, ashwagandha supplementation for 8 weeks increased estradiol concentrations by approximately 40% (Gopal et al., 2021). However, there has been no study conducted on post-menopausal women. It has been proposed that ashwagandha may increase estradiol via its GABA mimetic activity, which may stimulate LH secretion from the pituitary gland (Nasimi Doost Azgomi et al., 2018). Moreover, ashwagandha has demonstrated HPA axis-modulating effects (Lopresti et al., 2022), and since cortisol is inversely associated with sex hormones in both men and women (Pletzer et al., 2021), it is plausible that ashwagandha may have contributed to the increase in estradiol via this mechanism as well.

The significant increase in LH in men in the ashwagandha group compared to the placebo group is unexpected as there is generally a negative feedback relationship between FT and LH (Tilbrook and Clarke, 1995). However, as FT concentrations throughout the study remained within normal ranges (albeit low normal ranges), the magnitude of the increase in FT may not have triggered the negative feedback system. Moreover, aging is purported to impair the negative feedback of testosterone on the hypothalamus (Liu et al., 2009), hence the elevated LH concentrations of both groups of men (ashwagandha and placebo) throughout the duration of the study, but this theory requires further elucidation. Additionally, in vitro research demonstrated ashwagandha’s ability to upregulate gonadotropin-releasing hormone (GnRH) expression in hypothalamic cells, providing another potential mechanism for the concurrent elevated concentrations of FT and LH in the ashwagandha group (Kataria et al., 2015).

### Limitations and future directions

Despite some positive findings in the present study, the results need to be considered preliminary, with several factors affecting the robustness of the study. For example, due to the vast hormonal differences between the sexes, many blood results were analyzed by sex (Svechnikov and Soder, 2008). Thus, data from only 60 men and 60 women were available, making this study underpowered to identify all but large treatment effects. Future research should include larger sample sizes to more accurately analyze outcome measures with small-to-moderate effect sizes. The accuracy of results may have also been improved by increasing the frequency of measurement, with an additional assessment conducted midway through the study. Moreover, only a limited number of biological markers were examined in the present study. Future studies should include other measures of oxidative stress, such as myeloperoxidase and total antioxidant capacity, and more direct measures of inflammation such as C-reactive protein, tumor necrosis factor-alpha, and/or interleukin-6. These tests may be useful since ashwagandha has demonstrated anti-inflammatory and antioxidant properties (Atteeq, 2022; Kumar et al., 2022; Saggam et al., 2021). Also, indirect measures of HPA axis activation were used in the present study. Future studies may include hair cortisol concentrations to provide a chronic measure of circulating cortisol concentrations, multiple point cortisol measurements such as diurnal salivary cortisol or the cortisol awakening response, or the use of experimental stress procedures such as the Maastricht Acute Stress Test that provide a measure of HPA axis activity after exposure to an acute stressor (Ghassemi Nejad et al., 2022; Golden et al., 2013; Shin et al., 2011; Smeets et al., 2012). These investigations may be prudent since ashwagandha has potential HPA axis-modulating effects (Lopresti et al., 2022; Speers et al., 2021), which may contribute to its stress-relieving, anti-fatigue, and hormonal effects.

## Conclusion

In conclusion, supplementing with 200 mg of an ashwagandha extract (Witholytin®) twice daily for 12 weeks, in overweight or mildly obese men and women, aged 40–75 years, and experiencing high-stress levels and symptoms of fatigue, was associated with a significant reduction in perceived stress over time. However, stress reduction, as measured by the PSS (primary outcome measure), was not significantly different to those observed in the placebo group. Based on the findings from secondary and exploratory outcome measures, self-reported fatigue was reduced, and HRV increased over time compared to the placebo group. Men supplementing with the ashwagandha extract also experienced increases in FT and LH concentrations. However, compared to the placebo, ashwagandha supplementation was not associated with greater improvements in other self-report measures of stress and general well-being, or other measured blood markers. Based on these results, a further examination into populations that may benefit from ashwagandha supplementation will be important, as its stress-reducing effects may differ based on age, sex, BMI status, and other comorbidities. Moreover, the anti-fatigue effects of ashwagandha require further examination in clinical trials as fatigue is a symptom prevalent across many mental and physical conditions and can significantly impact quality of life. Ashwagandha supplementation was well-tolerated with no major self-reported adverse reactions and no changes in anthropometric measures (BMI, WC, and WHR) blood pressure, and safety blood makers comprising the liver function test, full blood count, and renal function. To help understand the benefits of ashwagandha supplementation and potential mechanisms of action, further investigation in robust, adequately powered clinical trials will be required.

## Supplemental Material

sj-docx-1-jop-10.1177_02698811231200023 – Supplemental material for Exploring the efficacy and safety of a novel standardized ashwagandha (Withania somnifera) root extract (Witholytin®) in adults experiencing high stress and fatigue in a randomized, double-blind, placebo-controlled trial Click here for additional data file. Supplemental material, sj-docx-1-jop-10.1177_02698811231200023 for Exploring the efficacy and safety of a novel standardized ashwagandha (Withania somnifera) root extract (Witholytin®) in adults experiencing high stress and fatigue in a randomized, double-blind, placebo-controlled trial by Stephen J Smith, Adrian L Lopresti and Timothy J Fairchild in Journal of Psychopharmacology
