# Supplementary material for: Exploring the efficacy and safety of a novel standardized ashwagandha (Withania somnifera) root extract (Witholytin®) in adults experiencing high stress and fatigue in a randomized, double-blind, placebo-controlled trial
Source: J Psychopharmacol. 2023 Sep 23;37(11):1091–104. doi: 10.1177/02698811231200023 (PMC10647917; doi:10.1177/02698811231200023)
Supplement: sj-docx-1-jop-10.1177_02698811231200023 – Supplemental material for Exploring the efficacy and safety of a novel standardized ashwagandha (Withania somnifera) root extract (Witholytin®) in adults experiencing high stress and fatigue in a randomized, double-blind, placebo-controlled trial [file sj-docx-1-jop-10.1177_02698811231200023.docx]

**SUPPLEMENTARY FILE**

**Exploring the efficacy and safety of a novel standardized Ashwagandha (Withania somnifera) root extract (Witholytin^®^) in adults experiencing high stress and fatigue in a randomized, double-blind, placebo-controlled trial**

**Author names:**

Stephen J Smith^1,2^, PhD candidate, [steve@clinicalresearch.com.au](mailto:steve@clinicalresearch.com.au)

Adrian L Lopresti^1,2^, PhD, [adrian@clinicalresearch.com.au](mailto:adrian@clinicalresearch.com.au)

Timothy J Fairchild^2^, PhD, [t.fairchild@murdoch.edu.au](mailto:t.fairchild@murdoch.edu.au)

**Author affiliations:**

^1^Clinical Research Australia, Perth, Western Australia, 6023, Australia

^2^College of Science, Health, Engineering and Education, Murdoch University, Perth, Western Australia, 6150, Australia

**Corresponding author:**

Stephen J Smith

Clinical Research Australia (CRA)

38 Arnisdale Rd Duncraig WA 6023

+61 (08) 9448 7376

[steve@clinicalresearch.com.au](mailto:steve@clinicalresearch.com.au)

## Blood sample analyses

### Collection of blood samples for analyses

Blood samples for plasma concentrations of TT, FT, oestradiol, LH, and DHEA-S were collected at baseline and week 12. Blood samples were collected in the morning between 8:00 am and 12:00 pm, in a fasted, non-exercised state, with alcohol being avoided the evening prior to providing the sample. The samples were collected in Ethylenediaminetetracetic acid (EDTA) tubes. The tubes were centrifuged at 1500xg for 15 minutes to separate the plasma from the red blood cells, with the plasma being removed from the EDTA tubes and placed in cryo-tubes, which were then stored in a -80° freezer for future analysis. On the day of analysis, samples were thawed, and an aliquot was used for measurement of TT, FT, oestradiol, LH, DHEA-S, and MDA. All analytes were measured in duplicate according to the manufacturer’s instructions. Assay kit performance characteristics are detailed below:

### Total Testosterone (TT)

On the day of the assay, plasma samples were thawed, and total testosterone measured using the commercially available LDN Testosterone ELISA kit (LDN Immunoassays and Services, Nordhorn, Germany) according to manufacturer instructions. The Testosterone ELISA kit is a solid phase competitive ELISA in which TT in the test sample competes for binding to an immobilised anti-testosterone monoclonal antibody with testosterone conjugated to horseradish peroxidase (HRP). After incubation at room temperature and several washes to remove all unbound complexes, bound HRP conjugated TT was incubated with the HRP substrate tetramethylbenzidine (TMB). The colourimetric reaction was stopped by the addition of 0.5M H_2_SO_4_ and the optical density (OD) of the resulting yellow solution measured at 450nm. The amount of TT in the test sample was inversely proportional to the yellow colour generated. The concentration of TT in samples was calculated by interpolation using 4-parameter non-linear regression curve fit software. Intra-Assay and Inter-Assay CV% was 3.6% and 7.1% respectively.

The minimum detectable level of TT using this assay kit was determined to be 0.083 ng/ml. This ELISA kit was specific for testosterone and showed 12.9% cross-reactivity with dihydrotestosterone (DHT), 3.3% cross reactivity to 11β-hydroxytestosterone and 19-nortestosterone, 0.8% cross reactivity to 5α-dihydrotestosterone and androstenedione, and negligible cross reactivity with all other steroid hormones tested.

### Free Testosterone (FT)

On the day of assay, plasma samples were thawed, and FT measured using the commercially available LDN Free Testosterone ELISA kit ^2nd Generation^ (LDN Immunoassays and Services, Nordhorn, Germany) according to manufacturer instructions. The Free Testosterone ELISA kit is a solid phase competitive ELISA in which FT in the test sample competes for binding to an immobilised anti-FT polyclonal antibody with FT conjugated to HRP. After incubation at 37^o^C and several washes to remove all unbound complexes, bound HRP conjugated FT was incubated with the HRP substrate TMB. The colourimetric reaction at 37^o^C was stopped by the addition on 0.5M H_2_SO_4_ and the OD of the resulting yellow solution measured at 450nm. The amount of FT in the test sample was inversely proportional to the yellow colour generated. Concentration of FT in samples was calculated by interpolation using 4-parameter non-linear regression curve fit software. Intra-Assay and Inter-Assay CV% is 6.0% and 8.8% respectively.

Note that testosterone circulates in the blood bound mostly with high affinity to sex hormone binding globulin (SHBG) and with lower affinity to albumin, and approximately 2-3% of total testosterone circulates in the unbound free form. According to the manufacturer, this kit utilises a highly specific rabbit anti-testosterone polyclonal antibody at a low binding capacity to keep minimum disturbances of the testosterone-binding protein equilibrium. Other components in the assay are also optimised to not alter the original FT concentration.

The minimum detectable level of FT using this assay kit was determined to be 0.018 pg/ml. This ELISA kit was specific for FT and showed 3.5% cross-reactivity with 5α-dihydrotestosterone, and negligible cross reactivity with all other steroid hormones tested.

### Dehydroepiandrosterone sulfate (DHEA-S)

On the day of assay, plasma samples were thawed, and DHEA-S measured using the commercially available LDN DHEA-S ELISA kit (LDN Immunoassays and Services, Nordhorn, Germany) according to manufacturer instructions. The DHEA-S ELISA kit is a solid phase competitive ELISA in which DHEA-S in the test sample competes for binding to an immobilised anti-DHEA-S polyclonal antibody with DHEA-S conjugated to HRP. After incubation at room temperature and several washes to remove all unbound complexes, bound HRP conjugated DHEA-S was incubated with the HRP substrate TMB. The colourimetric reaction was stopped by the addition of 0.5M H_2_SO_4_ and the OD of the resulting yellow solution measured at 450nm. The amount of DHEA-S in the test sample was inversely proportional to the yellow colour generated. The concentration of DHEA-S in samples was calculated by interpolation using 4-parameter non-linear regression curve fit software. Intra-Assay and Inter-Assay CV% was 9.3% and 9.2% respectively.

The minimum detectable level of testosterone using this assay kit was determined to be 5.0 ng/ml. This ELISA kit was specific for DHEA-S and showed 16.0% cross-reactivity with Androsterone, 1.7% to Androstenedione, 0.9% to Testosterone, 0.6% to Progestrone and Dihydrotestosterone and 0.5% to Cortisol.

### Oestradiol

On the day of assay, plasma samples were thawed, and oestradiol measured using the commercially available LDN Estradiol ELISA kit (LDN Immunoassays and Services, Nordhorn, Germany) according to manufacturer instructions. The Estradiol ELISA kit is a solid phase competitive ELISA in which oestradiol in the test sample competes for binding to an immobilised anti-oestradiol polyclonal antibody with oestradiol conjugated to HRP. After incubation at room temperature and several washes to remove all unbound complexes, bound HRP conjugated oestradiol was incubated with the HRP substrate TMB. The colourimetric reaction was stopped by the addition of 0.5M H_2_SO_4_ and the OD of the resulting yellow solution measured at 450nm. The amount of oestradiol in the test sample was inversely proportional to the yellow colour generated. The concentration of oestradiol in samples was calculated by interpolation using 4-parameter non-linear regression curve fit software. Intra-Assay and Inter-Assay CV% was 9.0% and 10.9% respectively.

The minimum detectable level of oestradiol using this assay kit was determined to be 10.6 pg/ml. This ELISA kit was specific for oestradiol and showed 6.9% cross-reactivity with estrone, 2.3% cross reactivity to estriol, 3.7% cross reactivity to fulvestrant, and negligible cross reactivity with all other steroid hormones tested.

### Luteinising Hormone (LH)

On the day of assay, plasma samples were thawed, and LH was measured using a commercially available ELISA kit (Demeditec Diagnostics, Kiel, Germany) according to manufacturer instructions. The LH ELISA kit is a solid phase sandwich ELISA in which LH in the test sample is captured by an immobilised anti-LH monoclonal antibody and sandwiched by an anti-LH monoclonal antibody conjugated to HRP. After incubation at room temperature and several washes to remove all unbound complexes, bound HRP conjugated antibody was incubated with the HRP substrate TMB. The colourimetric reaction was stopped by the addition of 0.5M H_2_SO_4_ and the OD of the resulting yellow solution was measured at 450nm. The amount of LH in the test sample was directly proportional to the yellow colour generated. The concentration of LH in samples was calculated by interpolation using 4-parameter non-linear regression curve fit software. The standards in this assay were calibrated against WHO 2^nd^ International Standard for LH IRP (80/552). Intra-Assay and Inter-Assay CV% were 5.6% and 6.2% respectively.

The minimum detectable level of LH using this assay kit was determined to be 1.27 mIU/ml. This ELISA kit was specific for LH and showed 5.2% cross-reactivity with human chorionic gonadotropin (WHO 1^st^ IRP 75/537), 3.0% cross reactivity with TSH (WHO 2^nd^ IRP 80/558), and 2.5% cross reactivity with follicle-stimulating hormone (WHO 1^st^ IRP 68/40).

### Malondialdehyde (MDA)

On the day of assay, plasma samples were thawed, and MDA was measured using a commercially available MDA ELISA kit (Wuhan Fine Biotech, Wuhan, China) according to manufacturer instructions. The MDA ELISA kit is a solid phase competitive ELISA in which MDA in the test sample competes with microtiter well immobilised MDA for binding to Biotinylated anti-MDA antibody. After incubation at 37^o^C and several washes to remove all unbound complexes, HRP-Streptavidin Conjugate was added and incubated at 37^o^C. Wells were washed again to remove unbound complexes and incubated with the HRP substrate TMB at 37^o^C. The colourimetric reaction was stopped by the addition of 0.5M H_2_SO_4_ and the OD of the resulting yellow solution measured at 450nm. The amount of MDA in the test sample was inversely proportional to the yellow colour generated. The concentration of MDA in samples was calculated by interpolation using 4-parameter non-linear regression curve fit software. Intra-Assay and Inter-Assay CV% was 3.6% and 7.1% respectively.

The minimum detectable level of MDA using this assay kit was determined to be 4.69 ng/ml. According to the manufacturer this ELISA kit is highly specific for MDA and shows no significant cross reactivity or interference between MDA and its analogues.

### Supplementary Table 1. Normative data on sex hormones concentrations

| **Testosterone (ng/mL)** | | **Range** |
| --- | --- | --- |
| Males | | 2 - 6.9 |
| Females | | 0.26 - 1.22 |
| **Free Testosterone (pg/mL)** | | **Range** |
| Males | 20-39 yrs | 9.1 - 32.2 |
|  | 40-59 yrs | 5.7 - 30.7 |
|  | > 60 yrs | 5.9 - 27.0 |
| Females | 20-39 yrs | 0.1 - 6.3 |
|  | 40-59 yrs | 0.2 - 4.2 |
|  | > 60 yrs | 0.5 3.9 |
| **DHEA-S (ug/mL)** | | **Range** |
| Males | | 0.39 - 4.63 |
| Females | | 0.46 - 2.75 |
| Post-menopausal Females | | 0.48 - 2.08 |
| **Oestradiol (pg/mL)** | | **Range** |
| Males | | 30.1 - 68.1 |
| Females | follicular phase | 28.1 - 178.1 |
|  | ovulation | 51.2 - 549.0 |
|  | luteal phase | 33.6 - 250.9 |
|  | post-menopausal | 18.4 - 64.0 |
| **LH (mIU/mL)** | | **Range** |
| Males | | 3 - 12 |
| Females | follicular phase | <=20 |
|  | luteal phase | <=20 |
|  | LH Surge | 20 - 200 |
|  | post-menopausal | 20 - 100 |

### Supplementary Table 2. Change in Body Composition Measures and Grip Strength (Men only; estimated marginal means)

|  |  | Ashwagandha (n=30) | | | Placebo (n=30) | | | p-value^b^ |
| --- | --- | --- | --- | --- | --- | --- | --- | --- |
|  |  | Week 0 | Week 12 | p-value^a^ | Week 0 | Week 12 | p-value^a^ |  |
| Body Mass Index | Mean | 29.79 | 29.55 | .301 | 29.79 | 29.65 | .538 | .763 |
|  | SE | 0.09 | 0.22 |  | 0.07 | 0.20 |  |  |
| Waist Circumference (cm) | Mean | 104.60 | 105.13 | .359 | 106.16 | 105.83 | .628 | .336 |
|  | SE | 1.11 | 1.13 |  | 0.56 | 0.62 |  |  |
| Waist-to-Hip Ratio | Mean | 0.96 | 0.96 | .402 | 0.97 | 0.97 | .391 | .230 |
|  | SE | 0.01 | 0.01 |  | 0.01 | 0.01 |  |  |
| Grip Strength (kg of pressure) | Mean | 47.16 | 48.04 | .117 | 50.83 | 52.11 | .054 | .713 |
|  | SE | 1.82 | 1.61 |  | 1.39 | 1.52 |  |  |

Results (estimated means) are generated from generalized mixed-effects models adjusted for age and BMI. ^a^P-values are generated from repeated measures generalized mixed-effects models adjusted for age and BMI (time effects baseline and week 12). ^b^P-values are generated from repeated measures generalized mixed-effects models adjusted for age and BMI (time x group interaction).

### Supplementary Table 3. Change in Body Composition Measures and Grip Strength (Women only; estimated marginal means)

|  |  | Ashwagandha (n=30) | | | Placebo (n=30) | | | p-value^b^ |
| --- | --- | --- | --- | --- | --- | --- | --- | --- |
|  |  | Week 0 | Week 12 | p-value^a^ | Week 0 | Week 12 | p-value^a^ |  |
| Body Mass Index | Mean | 29.16 | 29.15 | .919 | 29.12 | 29.30 | .148 | .317 |
|  | SE | 0.04 | 0.14 |  | 0.048 | 0.12 |  |  |
| Waist Circumference (cm) | Mean | 92.89 | 92.55 | .808 | 92.42 | 89.36 | .286 | .395 |
|  | SE | 1.58 | 1.71 |  | 1.88 | 2.80 |  |  |
| Waist-to-Hip Ratio* | Mean | 0.86 | 0.86 | .953 | 0.86 | 0.83 | .198 | .313 |
|  | SE | 0.02 | 0.02 |  | 0.02 | 0.02 |  |  |
| Grip Strength (kg of pressure) | Mean | 26.15 | 27.29 | .006 | 26.67 | 26.92 | .611 | .162 |
|  | SE | 0.76 | 0.74 |  | 0.89 | 0.91 |  |  |

Results (estimated means) are generated from generalized mixed-effects models adjusted for age and BMI. ^a^P-values are generated from repeated measures generalized mixed-effects models adjusted for age and BMI (time effects baseline and week 12). ^b^P-values are generated from repeated measures generalized mixed-effects models adjusted for age and BMI (time x group interaction). * Waist-to-hip ratio sample size: Ashwagandha (n=26); Placebo (n=27)

### Supplementary Table 4. Change in safety blood measures

|  | Ashwagandha (n=60) | | | | Placebo (n=60) | | | | p-value^a^ | p-value^b^ |
| --- | --- | --- | --- | --- | --- | --- | --- | --- | --- | --- |
|  | Week 0 | | Week 12 | | Week 0 | | Week 12 | |  |  |
|  | Mean | SE | Mean | SE | Mean | SE | Mean | SE |  |  |
| **Full Blood Count** | | | | | | | | | | |
| Haemoglobin (g/L) | 139.72 | 1.59 | 139.26 | 1.44 | 142.24 | 1.41 | 143.17 | 1.47 | .291 | .744 |
| Red blood cells (x10*12/L) | 4.68 | 0.06 | 4.70 | 0.06 | 4.74 | 0.05 | 4.78 | 0.05 | .720 |  |
| Haematocrit | 0.42 | 0.00 | 0.42 | 0.00 | 0.43 | 0.00 | 0.43 | 0.00 | .363 |  |
| Mean cell volume (fL) | 89.41 | 0.52 | 89.07 | 0.48 | 90.11 | 0.44 | 90.24 | 0.42 | .087 |  |
| Mean cell haemoglobin (pg) | 29.93 | 0.22 | 29.70 | 0.21 | 30.04 | 0.16 | 30.00 | 0.15 | .173 |  |
| Mean cell haemoglobin concentration (g/L) | 334.94 | 1.45 | 333.33 | 1.45 | 333.70 | 1.25 | 332.65 | 1.14 | .858 |  |
| RBC distribution width (%) | 12.76 | 0.11 | 14.75 | 2.06 | 12.55 | 0.08 | 12.48 | 0.09 | .249 |  |
| White cell count (x10*9/L) | 6.22 | 0.21 | 6.24 | 0.24 | 6.73 | 0.19 | 6.47 | 0.21 | .295 |  |
| Neutrophils (x10*9/L) | 3.46 | 0.15 | 3.44 | 0.17 | 3.87 | 0.17 | 3.71 | 0.15 | .512 |  |
| Lymphocytes (x10*9/L) | 2.05 | 0.08 | 2.04 | 0.08 | 2.14 | 0.08 | 2.06 | 0.08 | .438 |  |
| Monocytes (x10*9/L) | 0.51 | 0.02 | 0.51 | 0.03 | 0.53 | 0.02 | 0.51 | 0.02 | .470 |  |
| Eosinophils (x10*9/L) | 0.13 | 0.02 | 0.14 | 0.02 | 0.13 | 0.02 | 0.13 | 0.02 | .946 |  |
| Basophils (x10*9/L) | 0.01 | 0.00 | 0.01 | 0.01 | 0.00 | 0.00 | 0.00 | 0.00 | .937 |  |
| Platelets (x10*9/L) | 248.26 | 8.18 | 255.04 | 9.31 | 258.15 | 6.77 | 258.98 | 8.76 | .299 |  |
| Mean platelet volume (fL) | 10.76 | 0.12 | 10.71 | 0.12 | 10.58 | 0.12 | 10.65 | 0.13 | .094 |  |
| **Liver Function test** | | | | | | | | | | |
| Aspartate transaminase (mmol/L) | 28.11 | 1.07 | 27.35 | 0.97 | 29.70 | 2.34 | 26.85 | 0.83 | .361 | .891 |
| Alanine aminotransferase (U/L) | 30.59 | 2.21 | 29.22 | 1.76 | 32.81 | 2.82 | 30.15 | 1.91 | .563 |  |
| Alkaline Phosphatase (U/L) | 76.17 | 2.71 | 74.76 | 2.59 | 78.37 | 2.69 | 77.07 | 2.82 | .956 |  |
| Gamma-glutamyl transferase (U/L) | 21.74 | 2.01 | 19.65 | 1.73 | 26.74 | 4.16 | 24.30 | 2.60 | .870 |  |
| Total Protein (g/L) | 67.87 | 0.55 | 67.96 | 0.51 | 67.04 | 0.47 | 67.65 | 0.47 | .368 |  |
| Bilirubin (umol/L) | 10.63 | 0.58 | 10.43 | 0.68 | 10.94 | 0.71 | 10.24 | 0.73 | .494 |  |
| Globulin (g/L) | 28.04 | 0.43 | 28.39 | 0.45 | 27.63 | 0.40 | 28.46 | 0.43 | .265 |  |
| Albumin (g/L) | 39.83 | 0.37 | 39.57 | 0.30 | 39.41 | 0.32 | 39.19 | 0.33 | .933 |  |
| **Renal Function** | | | | | | | | | | |
| Urea (mmol/L) | 5.76 | 0.20 | 5.70 | 0.16 | 5.81 | 0.21 | 5.81 | 0.18 | .796 | .817 |
| Creatinine (umol/L) | 77.94 | 1.89 | 76.70 | 1.86 | 77.87 | 2.58 | 78.11 | 2.08 | .885 |  |
| Estimated glomerular filtration rate (mL/min/1.73m^2^) | 82.90 | 1.52 | 83.23 | 1.39 | 81.94 | 1.33 | 82.28 | 1.26 | .547 |  |
| Sodium (mmol/L) | 139.58 | 0.39 | 139.61 | 0.32 | 140.35 | 0.34 | 140.19 | 0.25 | .467 |  |
| Potassium (mmol/L) | 4.32 | 0.04 | 4.36 | 0.03 | 4.31 | 0.04 | 4.39 | 0.04 | .428 |  |
| Chloride (mmol/L) | 104.56 | 0.34 | 104.65 | 0.30 | 105.72 | 0.27 | 105.28 | 0.29 | .279 |  |
| Bicarbonate (mmol/L) | 27.66 | 0.34 | 27.00 | 0.31 | 27.26 | 0.31 | 27.30 | 0.25 | .355 |  |
| Anion Gap (mmol/L) | 11.55 | 0.35 | 12.22 | 0.23 | 11.57 | 0.33 | 11.93 | 0.28 | .642 |  |

^a^Based on change scores (baseline to week 12) using a Multivariate ANOVA (covariates age and BMI), univariate results; ^b^Based on change scores (baseline to week 12) using a Multivariate ANOVA (covariates age and BMI)
